# High-resolution melting PCR assay, applicable for diagnostics and screening studies, allowing detection and differentiation of several *Babesia* spp. infecting humans and animals

**DOI:** 10.1007/s00436-017-5576-x

**Published:** 2017-08-10

**Authors:** Wioletta Rozej-Bielicka, Aleksander Masny, Elzbieta Golab

**Affiliations:** Department of Parasitology, National Institute of Public Health – National Institute of Hygiene, Warszawa, Poland

**Keywords:** *Babesia*, *Babesia microti*, *Babesia divergens*, *Babesia venatorum*, *Babesia canis*, Real-time PCR, Multiplex-PCR, HRM, High-resolution melting, Melting curve, DNA detection, Species identification, Single-tube PCR, 18S rRNA, Universal primers

## Abstract

The goal of the study was to design a single tube PCR test for detection and differentiation of *Babesia* species in DNA samples obtained from diverse biological materials. A multiplex, single tube PCR test was designed for amplification of approximately 400 bp region of the *Babesia* 18S rRNA gene. Universal primers were designed to match DNA of multiple *Babesia* spp. and to have low levels of similarity to DNA sequences of other intracellular protozoa and *Babesia* hosts. The PCR products amplified from *Babesia* DNA isolated from human, dog, rodent, deer, and tick samples were subjected to high-resolution melting analysis for *Babesia* species identification. The designed test allowed detection and differentiation of four *Babesia* species, three zoonotic (*B. microti*, *B. divergens*, *B. venatorum*) and one that is generally not considered zoonotic—*Babesia canis*. Both detection and identification of all four species were possible based on the HRM curves of the PCR products in samples obtained from the following: humans, dogs, rodents, and ticks. No cross-reactivity with DNA of *Babesia* hosts or *Plasmodium falciparum* and *Toxoplasma gondii* was observed. The lack of cross-reactivity with *P. falciparum* DNA might allow using the assay in endemic malaria areas. The designed assay is the first PCR-based test for detection and differentiation of several *Babesia* spp. of medical and veterinary importance, in a single tube reaction. The results of the study show that the designed assay for *Babesia* detection and identification could be a practical and inexpensive tool for diagnostics and screening studies of diverse biological materials.

**Electronic supplementary material:**

The online version of this article (doi:10.1007/s00436-017-5576-x) contains supplementary material, which is available to authorized users.

## Introduction

*Babesia* spp*.* are tick-transmitted protozoan hemoparasites, considered to be the second most commonly found parasites in the blood of mammals (Schnittger et al. 2012). Infections with *Babesia* can be fatal in humans, cattle, and companion animals (Schnittger et al. 2012). Babesiosis causes economic loss in animal production in the USA, Australia, and South America (Canever et al. 2014). Furthermore, *Babesia* infections pose a public health threat since the parasite can be transmitted not only through tick bites but also by transfusion of blood and blood products. However, there still are no standardized procedures of screening blood for *Babesia* even in such developed countries as the USA, where cases of babesiosis related to blood transfusion have been recorded (Bish et al. 2015; Leiby 2006; Levin and Krause 2016).

Four *Babesia* species and a few strains with uncertain taxonomic position have been recognized as pathogenic for humans so far. In North America, the main causative agent of human babesiosis is *B. microti* whereas in Europe, most of the described human autochthonous infections were caused by *B. divergens* and *B. venatorum* (Haapasalo et al. 2010; Zintl et al. 2003). In Asia, the public health risk associated with *B. microti* and *B. venatorum* infections has been recognized recently (Zhou et al. 2013, 2014). There are no official recommendations on laboratory diagnosis of *Babesia* infection both in the USA and in European countries (Hildebrandt et al. 2013; Levin and Krause 2016). Definitive diagnosis in most cases still depends upon finding of parasites on blood smear examination. The commercial serological and molecular tests used in the routine diagnostics of human babesiosis are not standardized. Most often, serological tests, designed mainly for infections with *B. microti*, are used in medical laboratories. However, clinicians in their practice have problems with interpretation of these test results since the positive results may correspond to: present, past, or no infection (Prince et al. 2010). False negative as well as false positive results due to cross-reactivity with other intracellular parasites such as *Toxoplasma gondii* and *Plasmodium* have also been observed in ELISA tests (Hildebrandt et al. 2013). However, the risk of confusing of *Plasmodium* and *Babesia* also occurs when microscopy is used for blood smear examination (Zhou et al. 2013).

It was shown that PCR for amplification 18S rRNA fragments can be more sensitive than microscopy for detection of *B. microti* infection (Teal et al. 2012; Wang et al. 2015a, b). Recently, numerous PCR-based assays for detection of *Babesia*, important from medical and veterinary points of view, have been developed (Li et al. 2015; Teal et al. 2012; Wang et al. 2015b). However, multiplex PCR for simultaneous detection and differentiation of several *Babesia* that infect humans has not been described to date, even though the need for such a tool has been expressed (Hildebrandt et al. 2013). The objective of this study was to design a single tube PCR test for detection and differentiation of *Babesia* species in DNA samples obtained from diverse biological materials.

## Materials and methods

### Biological material

Clinical and environmental DNA samples were tested: human, dog, *Microtus oeconomus*, *Caproleus caproleus*, and *Ixodes ricinus* (Table 1). Human clinical samples tested for malaria, babesiosis, and toxoplasmosis originated from routine diagnostics of the Diagnostic Laboratory of the Department of Parasitology NIPH-NIH. Obtaining the patients’ consent for use of their samples for research purposes is a standard procedure in the NIPH-NIH Diagnostics Laboratories. A single human blood sample positive for *Babesia venatorum* was collected during project NN404520038 financed by the Ministry of Science and Higher Education. The granted study was approved by the Bioethics Commission in NIPH-NIH (Sadkowska-Todys et al. 2015). The dog blood samples collected for routine diagnostics were obtained from the patients of the Antecedo (Rafal Stachurski and Katarzyna Stachurska) veterinary clinic. Blood smear examination was used by veterinarians to confirm *Babesia* infection. Roe deer (*Caproleus caproleus*) spleen sample was obtained through the courtesy of Prof. Bogumila Skotarczak from the University of Szczecin (Sawczuk et al. 2005). *Microtus oeconomus* and *Caproleus caproleus* blood samples were obtained through the courtesy of Prof. Grzegorz Karbowiak from the Polish Academy of Sciences, Warsaw. The latter samples were examined by microscopy (thick blood smears). Tick DNA samples originated from the Department’s collection.

**Table 1:** Origin of the control DNA samples used in the study

Source of DNA	Control DNA (no. of tested samples)/status
Human:	
Blood	*B. venatorum* asymptomatic infection (1) / P
	*Plasmodium falciparum* infection (1)/N
	*Babesia* spp. negative (5)/N
Amniotic fluid	*T. gondii* infection (1)/N
	*T. gondii* negative (1)/N
Dog blood	*B. canis* symptomatic infection (10)/P
	*Babesia* spp. negative (1)/N
Blood of *Microtus oeconomus*	*B. microti* infection (5)/P
	*Babesia* spp. negative (1)/N
Spleen of *Caproleus caproleus*	*B. divergens* infection (1)/P
Blood of *Caproleus caproleus*	*Babesia* spp. negative/N
*Ixodes ricinus* adult males DNA	*B. canis* infection (1)/P
	*Babesia* spp. negative (1)/N

Source of DNA: The source of biological material used for DNA isolation. Control DNA sample–sample description and the number of samples (indicated in brackets). Status: *N Babesia* negative, *P Babesia* positive. In multiplex HRM PCR, all *Babesia* postitive samples were positive, all *Babesia* negative samples were negative

### DNA extraction

The DNA from animal blood and ticks was isolated according to the silica guanidinium protocol (Boom et al. 1999) with modifications for whole blood and arthropod samples (Masny et al. 2011, 2016). The DNA from *C. caproleus* spleen was isolated using DNeasy Blood & Tissue Kit (Qiagen) according to the manufacturer’s instructions.

### Control sample selection

The status of all samples, positive or negative for *Babesia*, was confirmed by PCR with universal primer pair for *Babesia* and *Theileria* amplification: Bab-GF2 (5′-GYYTTGTAATTGGAATGATGG-3′) and Bab-GR2 (5′-CCAAAGACTTTGATTTCTCTC-3′) (Bonnet et al. 2007) with minor modifications required to adjust the reaction conditions to the reagents used; Hot Star Taq DNA PCR 0.625 U polymerase, 1× PCR buffer (Qiagen), 1 μl in total volume of 25 μl, were used, the PCR started with a polymerase activation phase at 94 °C for 15 min, followed by 40 cycles at 94 °C for 60 s, 60 °C for 90 s, and 72 °C for 60 s. The PCR products were sequenced [GenBank: KP072001.1, KT844903.1, KT844904.1, KT844905.1, KT844906.1, KT844907.1, KT844908.1, KT844909.1, KT844910.1, KT844911.1, KT844912.1, KT869379.1], and the species of *Babesia* was determined based on the similarity to the genomic sequences deposited in GenBank.

### Sequence analysis and primer design for multiplex PCR HRM

Multiple sequence alignments were performed with ClustalX2 (Larkin et al. 2007) and MAFFT algorithm using Jalview software (Waterhouse et al. 2009) and subsequently were manually edited using SeaView (Gouy et al. 2010) and Jalview software packages. The primers were designed manually, based on the alignment of the following 18S rRNA *Babesia* nucleotide sequences obtained from GenBank: AJ439713.1, EF458228.1, FJ944822.1, FJ944823.1 (*B. divergens*); AY693840.1, EF413181.1, JX417370.1, KC470047.1, KC821597.1, KF410824.1, KF410825.1, KF410826.1, KF410827.1 (*B. microti*); KF724377.1, KJ663730.1, KM244044.1 (*B. venatorum* also described as *Babesia* sp. EU1) and AY048113.1 (*Babesia* sp. MO1). The three designed primers were universal for at least seven *Babesia* taxons (Table 2), five of them described to infect humans: *B. divergens*, *B. divergens-*like, *B. microti*, *B. venatorum*, *Babesia microti-*like.

**Table 2:** The list of 18 taxons (species and species-like) with perfect binding site of the universal reverse primer B-rev

Primer	Species based matching / similarity (%)																	
	*B. microti*	*B. microti-*like	*B. divergens*	*B. divergens-*like	*B. venatorum* ( *Babesia* sp. EU1)	*B. capreoli*	*B. odocoilei*	*B. canis*	*B. hongkongensis*	*B. vogeli*	*B. caballi*	*B. rossi*	*B. occultans*	*B. duncani*	*B. gibsoni*	*B. lengau*	*B. crassa*	*B. behnkei*
B-rev	23/23 (100%)	23/23 (100%)	23/23 (100%)	23/23 (100%)	23/23 (100%)	23/23 (100%)	23/23 (100%)	23/23 (100%)	23/23 (100%)	23/23 (100%)	23/23 (100%)	23/23 (100%)	23/23 (100%)	23/23 (100%)	23/23 (100%)	23/23 (100%)	23/23 (100%)	23/23 (100%)
B-BM	25/25 (100%)	25/25 (100%)	14/25 (56%)	17/25 (68%)	17/25 (68%)	17/25 (68%)	17/25 (68%)	15/25 (60%)	16/25 (64%)	15/25 (60%)	12/25 (48%)	16/25 (64%)	16/25 (64%)	22/25 (88%)	22/25 (88%)	21/25 (84%)	8/25 (32%)	7/25 (28%)
B-BDV	18/25 (72%)	17/25 (68%)	25/25 (100%)	25/25 (100%)	25/25 (100%)	25/25 (100%)	25/25 (100%)	23/25 (92%)	23/25 (92%)	23/25 (92%)	23/25 (92%)	22/25 (88%)	22/25 (88%)	14/25 (56%)	12/25 (48%)	20/25 (80%)	9/25 (36%)	8/25 (32%)

The level of similarity between the primer and the primer binding region is expressed as the number of identities and percentage similarity obtained using NCBI BLAST

Seven taxons have perfect binding sites for a pair of primers B-rev/B-BM (two taxons) or B-rev/B-BDV (five taxons). The underlined names belong to *Babesia* spp. for which experimental confirmation of multiplex PCR HRM amplification is available and all those species were amplified and differentiated by multiplex PCR HRM. The list of *Babesia* genomic sequences perfectly matching the primers B-Rev, B-BM, and B-BDV is provided in Table S1

**Fig. 1:**
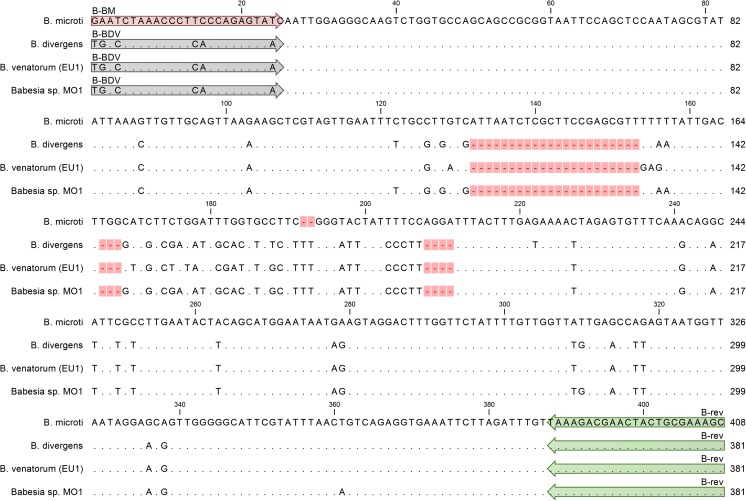
Multiple alignment of the 18S rRNA gene region amplified in multiplex HRM PCR. The reference sequence from *B. microti* is presented on the top of the alignment. The bases identical to those in the reference sequence are represented by dots in the sequences from the following: *B. divergens*, *B. venatorum* (EU1), *Babesia* sp. MO1. Only the bases that differentiate *B. divergens*, *B. venatorum* (EU1), *Babesia* sp. MO1 from the reference sequence are shown in the alignment as A, G, C, and T. The sequence of *B. microti* is the longest—408 bases versus 381 bases in case of sequences from the remaining three species, the gaps in those sequences are indicated by *dashes on a pink background*. Primer regions are indicated by *arrows* with the primer name above them. B-BM primer perfectly matches only the *B. microti* sequence. B-BDV primer perfectly matches sequences of *B. divergens*, *B. venatorum* (EU1), and *Babesia* sp. MO1, presented on the figure. B-BDV perfectly matches sequences of all four taxons. All four 18S rRNA gene regions, spanned by primer pairs B-BM/B-rev and B-BDV/B-rev, are polymorphic and have unique sequences

The forward primers were as follows: B-BM (5′-GAATCTAAACCCTTCCCAGAGTATC-3′), B-BDV (5′-TGACCTAAACCCTCACCAGAGTAAC-3′) and the universal reverse primer was B-rev (5′-GCTTTCGCAGTAGTTCGTCTTTA-3′). The primer pairs B-rev, B-BDV and B-rev, B-BM flanked 381 and 408 bp 18S rRNA gene fragments of *B. divergens*, *B. divergens-*like, *B. venatorum* and *B. microti*, *Babesia microti-*like, respectively (Fig. 1).

The range of primer specificity and potential of the primer cross-reactivity was estimated by NCBI BLAST search with default settings and relaxed settings (Masny et al. 2016).

Sanger sequencing of all samples sequenced in this study was performed by external service oligo.pl (Warsaw, Poland).

### Multiplex PCR HRM

The reaction mix for mPCR-HRM composition was as follows: 1× concentrated SsoFast™ EvaGreen® Supermix, 0.4 μM of each primer, and 2 μl of template DNA in total volume of 25 μl. The PCR was performed in touchdown format, the initial denaturation at 98 °C for 3 min was followed by 40 cycles of three step reaction: 1st step, 98 °C for 15 s; 2nd step, touchdown from 63 to 55 °C for 30 s (gradual reduction of the temperature by 0.2 °C in every consecutive cycle); 3rd step, 72 °C for 30 s. Signal was acquired in every cycle, in green channel, at the end of incubation at 72 °C. After completion of PCR, premelt conditioning was performed for 90 s with default settings and was followed by HRM in a temperature range from 70 to 90 °C, in 0.2 °C temperature increments, with incubation for 3 s at each step.

### Species differentiation

Genotype identification procedure was performed using Rotor Gene 6000 software–version 1.7 build 85 to discriminate *Babesia* spp. For establishing the confidence of identification, three replicates of HRM for each *Babesia* spp. were used. The threshold for species identification was set to 95% level of confidence. Difference plots of HRM curves were obtained with Rotor Gene 6000 software–version 1.7 build 85 to visualize inter-species differences between melting curves, each of the species was used as a reference, in separate analyses, for obtaining difference plots.

### Analytical sensitivity

The detection limit of mPCR-HRM performance and detection limit studies were performed on serial dilutions of *B. microti* 18S rRNA fragment, amplified with the primers CRYPTOF (5′-AACCTGGTTGATCCTGCCAGTAGTCAT-3′) and CRYPTOR (5′-AATGATCCTTCCGCAGGTTCACCTAC-3′). The primer pair CRYPTOF and CRYPTOR (Herwaldt et al. 2003) spans 1770 bp DNA fragment of *B. microti* 18S rRNA. The region amplified by mPCR-HRM is located within the 1770 bp fragment. The amplified 1770 bp 18S rRNA fragment was subjected to agarose gel electrophoresis and isolated from the agarose gel using the Gel-Out® kit (A&A Biotechnology). The concentration of the purified DNA fragment was measured by UV spectroscopy and 7 ten-fold; serial dilutions of the PCR product in water were prepared. Seven dilutions from 5 × 10^6^ copies/μl to 5 copies/μl were used as a template for mPCR-HRM. The mPCR-HRM reactions were performed in triplicate for each of the dilutions, and such independent reactions were performed in three independent PCR runs.

## Results

**Fig. 2:**
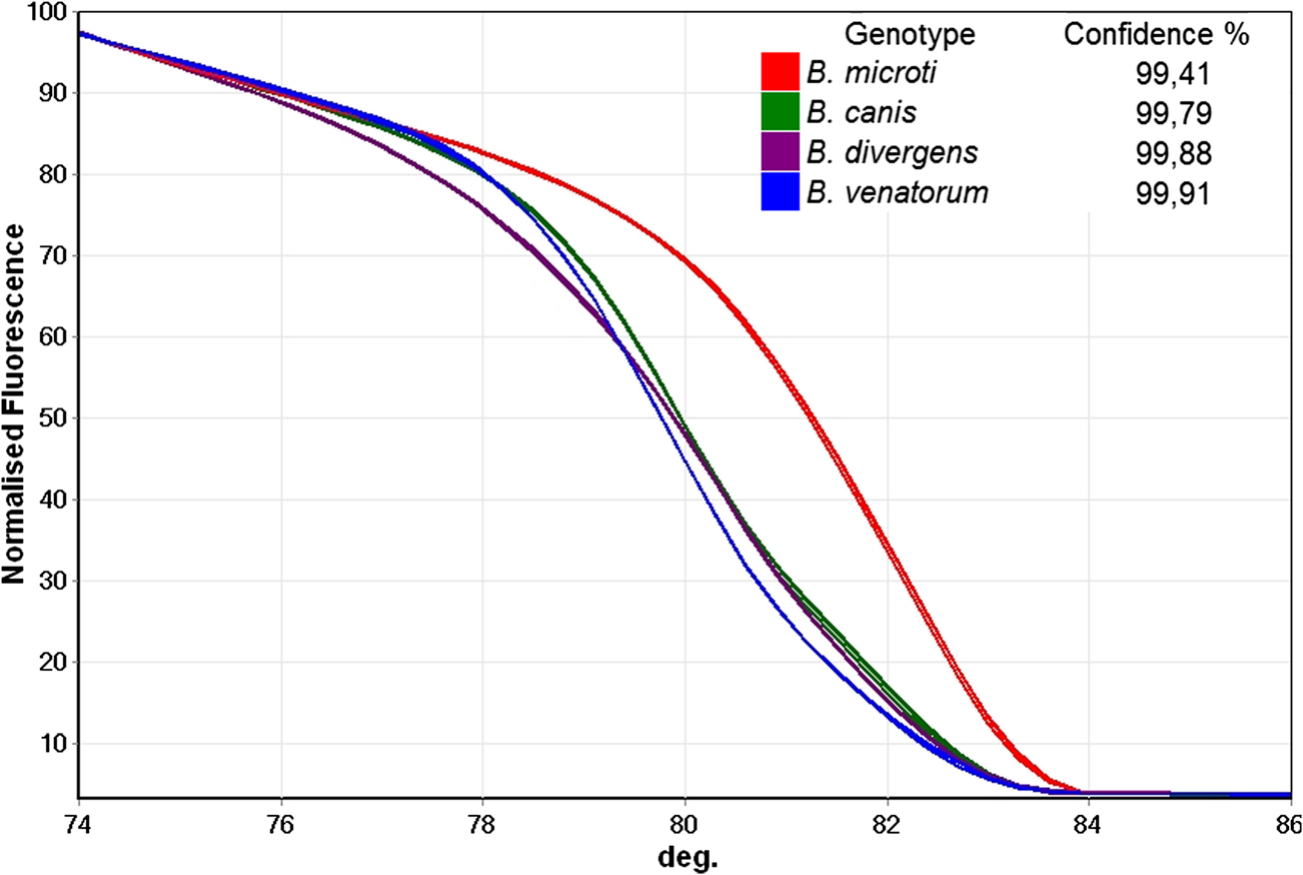
Differentiation of *Babesia* species by high-resolution melting curves. Melting curves—the plots of normalized fluorescence versus melting temperature obtained for four species. Average values of genotype assignment confidence (for the three repeats of experiments performed for each species) and the legend to the figure are presented on the graph

**Fig. 3:**
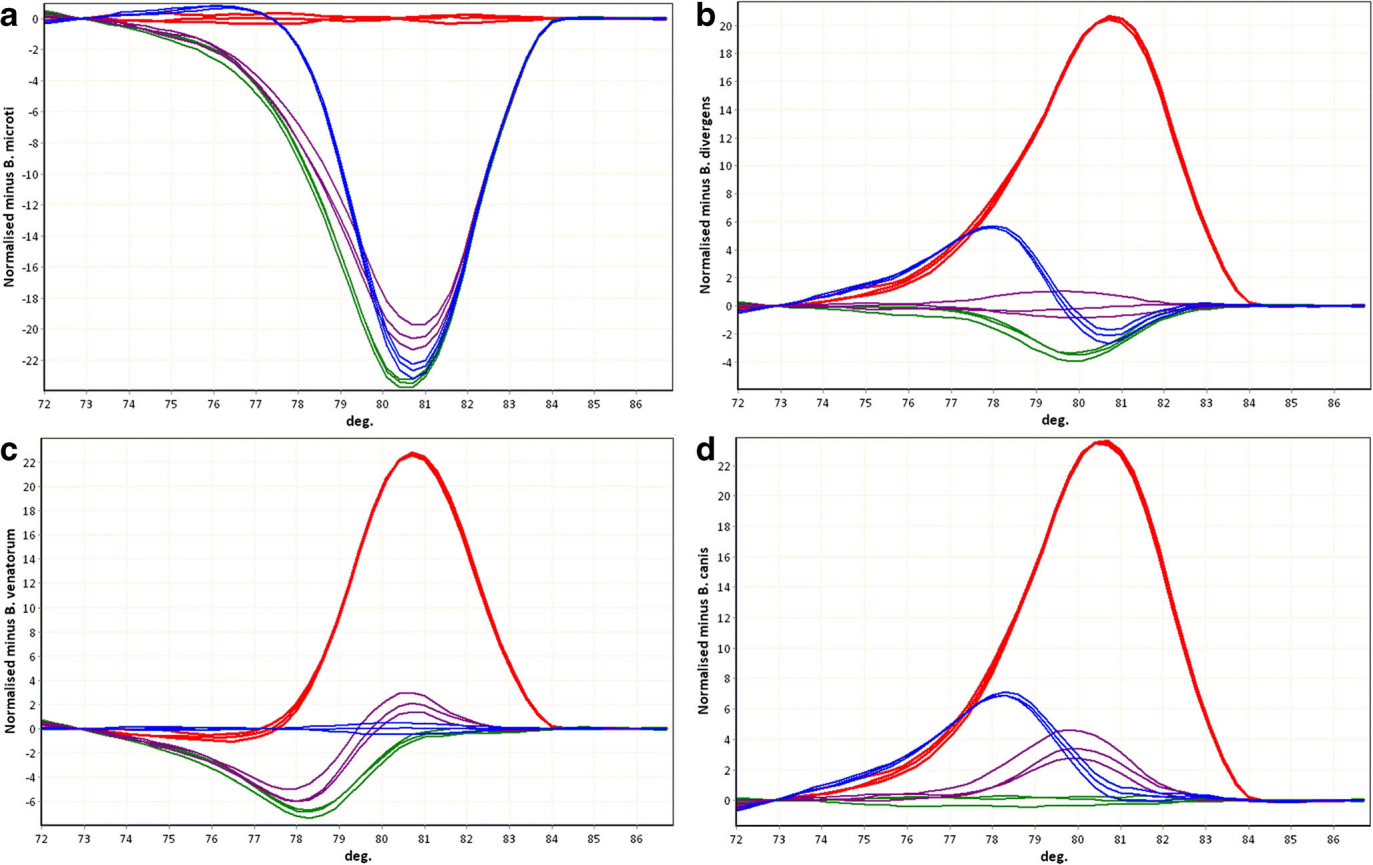
Babesia species discrimination by difference curves. *Red B. microti*, *purple B. divergens*, *blue B. venatorum*, *green B. canis*. The multiplex PCR products obtained from DNA of the following species were used as the references for difference curve plot. **a** *B. microti*. **b** *B. divergens*. **c** *B. venatorum*. **d** *B. canis*. Melting curves were automatically normalized and a reference curves were selected by the user. Values of each curve were automatically subtracted, by the software, from the values of the user selected reference curve and the results were presented on the plot. The more similar are two curves the closer the plotted line should be to a straight horizontal line (such as the repeats of the reference species curve on the plot of difference curves). The differences between the melting curves of the multiplex PCR products are emphasized on difference plots and observed as the differences in the shapes of the difference curves. Such curve shape differences correspond to the differences at the level of nucleotide sequence (only those sequence alterations that cause DNA melting temperature change). Differentiation of all four tested Babesia species was possible for each of the four species used as a reference—each species had unique difference curve shape

In multiplex PCR with forward primers B-BDV, B-BM, and B-rev reverse primer, DNA of *B. canis*, *B. divergens*, *B. venatorum*, and *B. microti* was amplified. Application of HRM curve analysis allowed discrimination of the four *Babesia* species both visually and using the automatic ‘genotype’ assignment feature of the software (Fig. 2 and Fig. 3).

**Fig. 4:**
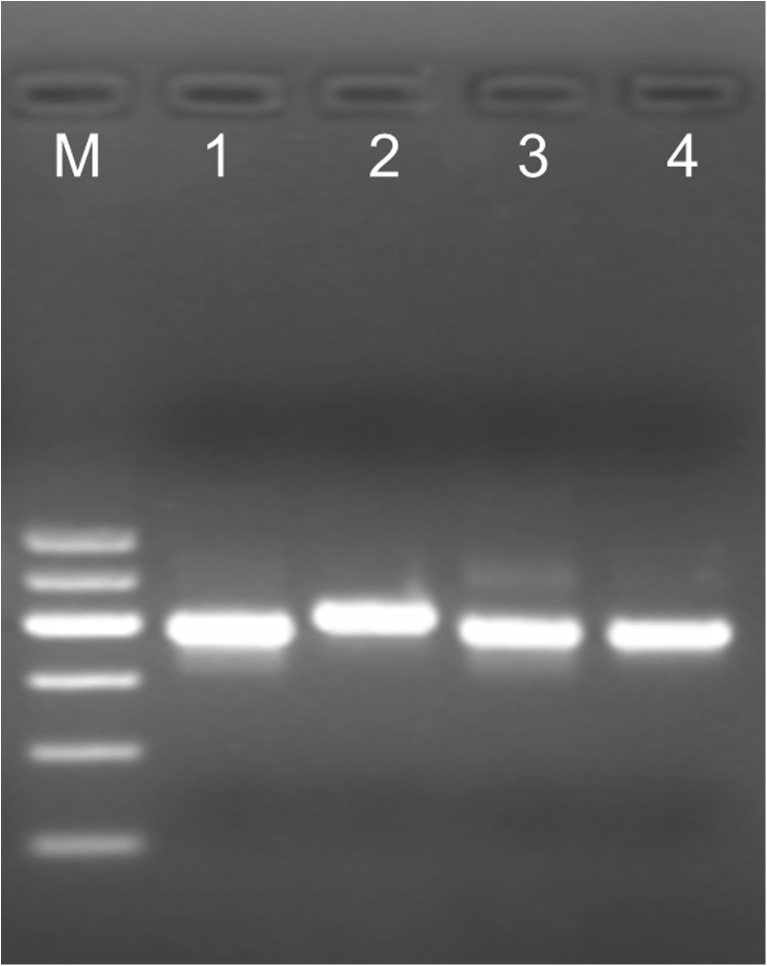
Gel electrophoresis of multiplex PCR HRM products. Agarose gel electrophoresis allowed discrimination between the four tested *Babesia* species. The mPCR-HRM products obtained for each species were separated in 3% agarose gel in the following order: (1) *B. canis*, (2) *B. microti*, (3) *B. divergens*, and (4) *B. venatorum*. M: 100 bp DNA ladder (GPB 600 bp DNA Ladder, GenoPlast)

Agarose gel electrophoresis allowed discrimination between *B. microti* and the three other species: *B. canis*, *B. divergens*, *B. venatorum*, 408 and 381 bp fragments, respectively (Fig. 4).

**Fig. 5:**
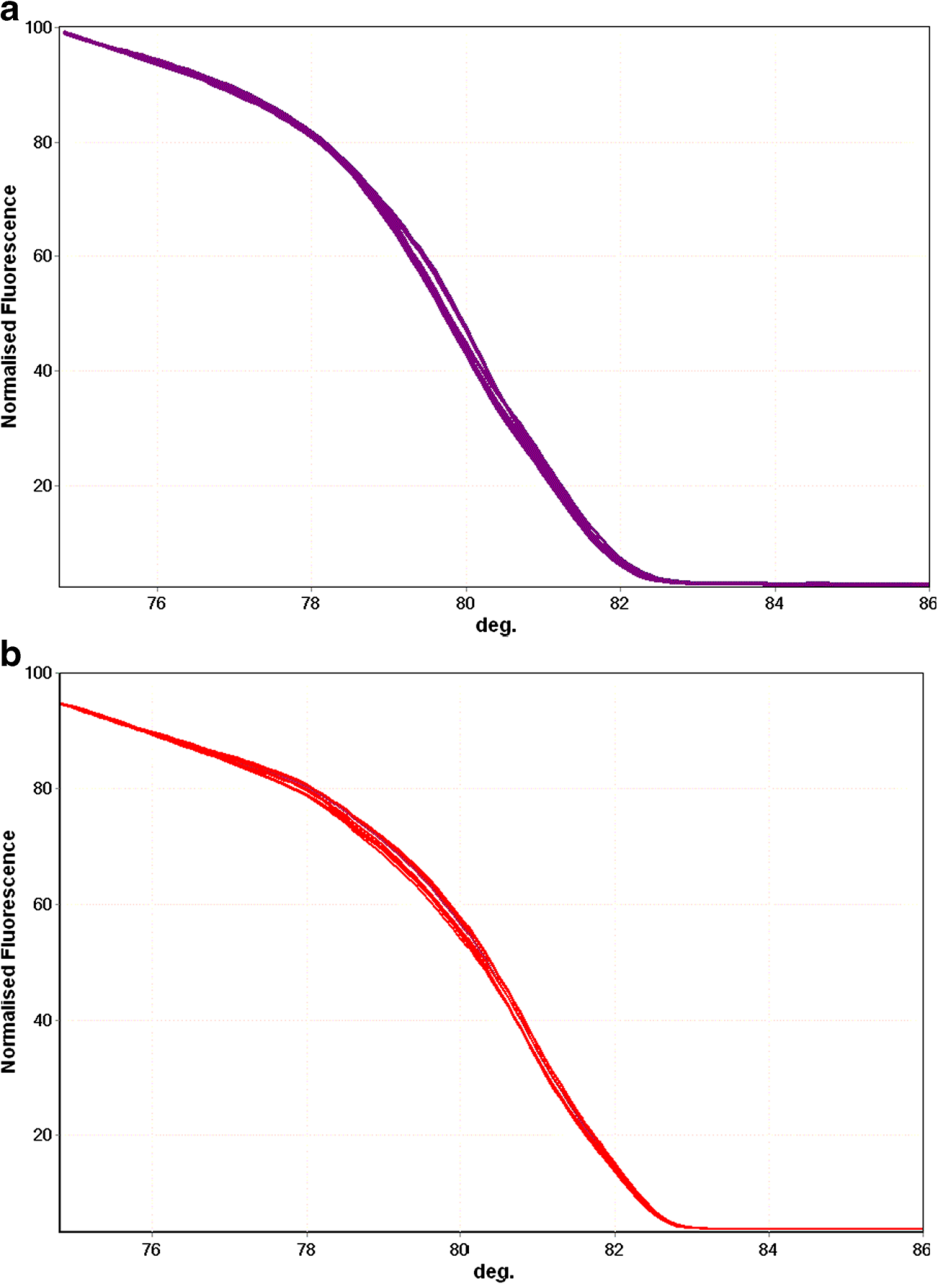
Sensitivity of detection. The HRM curves obtained for 7 tenfold dilutions (in triplicate) of target DNA from 5 copies to do 5 × 10^6^ copies for **a** *Babesia divergens* (*purple*) and **b** *Babesia microti* (*red*)

The mPCR-HRM detection limit of *B. microti* and *B. divergens* was at least 5 copies of 18S rRNA target sequences (Fig. 5), detected in 6 repeats, i.e., in the three samples in the two independent mPCR-HRM reactions with 100% positive results.

In silico analyses of mPCR-HRM specificity revealed the presence of perfect binding sites for the primer B-rev in at least 18 *Babesia* taxons and multiple perfect and imperfect binding sites for the forward primers (Table 2). Perfect binding sites for primer pairs, B-BDV/B-rev and B-BM/B-rev, were present in 5 and 2 *Babesia* species, respectively (Table 2). The primer pair B-BM/B-rev has perfect binding site in the genome of *Theileria* sp. HN1 [GenBank: FJ645726.1] (Table S2).

In silico no significant similarity was found between the designed primer pairs and the DNA of the following: human, dog, ticks, roe deer, ungulates, *Plasmodium falciparum*, and *Toxoplasma gondii*. The specificity was confirmed experimentally in mPCR-HRM with negative control DNA samples.

Ten blood samples from dogs with confirmed *B. canis* infection and 5 *Microtus oeconomus* blood samples with confirmed *B. microti* infection gave reproducible, positive results of mPCR-HRM species determination (Fig. S1).

## Discussion

The most important feature of the designed mPCR-HRM method is the reduction of the procedure of DNA detection and identification of several *Babesia* species to a single tube reaction.

The clinical course of babesiosis in immunocompromised patients is often severe, and the mortality rate in such cases may reach 20% (Levin and Krause 2016). In contrast, in adult immunocompetent persons, who can be blood donors, the course of *Babesia* infection is asymptomatic. In most such cases, low level parasitemia persists in blood for a few months from the day of the infection (Krause et al. 1998; Vannier and Krause 2012).

There has been growing concern about the risk of *B. microti* transmission by blood and blood derived products in the USA (Levin and Krause 2016; Simon et al. 2014). The discussions on the necessity of blood screening to prevent transfusion transmitted babesiosis and on optimal screening tools continue (Bish et al. 2015; Leiby 2006; Levin and Krause 2016; Simon et al. 2014). However, it is known that the screening costs should not exceed its benefit for public health (Levin and Krause 2016; Simon et al. 2014).

The designed multiplex PCR HRM method is a tool for detection of infections with at least three *Babesia* species pathogenic to humans, in a single tube assay, which might match economic criteria for babesiosis blood screening tests. The assay was proven to have no cross reactions with protozoan parasites *Toxoplasma gondii* and *Plasmodium falciparum* related to *Babesia*. Such a cross reaction may pose a problem in serology.

Ideally, the sensitivity of microscopy examination, still a “gold standard” in diagnostics of intraerythrocytic protozoa, should reach a detection limit of 10–50 parasites/μl of blood in a thick blood smear in a person with a normal red blood cell count (Guerin et al. 2002; Kamau et al. 2011; Moody 2002; Wang et al. 2015b). However, most laboratories require ten-fold higher parasitemia (100–500) to detect intraerythrocytic parasite infection (development of recommendations for the protection of short-stay travelers to malaria endemic areas: Memorandum from two WHO Meetings 1988; Milne et al. 1994; Wang et al. 2015b). Furthermore, the infected red blood cell count can vary depending on the person performing microscopy examination of the same blood sample (Wang et al. 2015b).

The sensitivity of most PCR methods for *B. microti* detection, employing DNA isolation procedures used routinely in blood diagnostics, was estimated to be within the range 1–30 parasites per one 1 μl of blood (Chan et al. 2013; Rollend et al. 2013; Wang et al. 2015a, b). The only method claimed to have higher sensitivity (Bloch et al. 2013) used a non-standard approach for DNA isolation which is not used in routine blood examination performed in medical diagnostics (Wang et al. 2015b). Our assay detects at least 5 copies of the *Babesia* spp. 18S rRNA fragment in 1 μl of solution added to PCR. The sensitivity was tested using the DNA of *B. microti* and *B. divergens* (Fig. 5); thus, both primer pairs used in mPCR-HRM were tested: B-BM/B-rev and B-BDV/B-rev, respectively. At least two copies of the target 18S rRNA are present in one *Babesia* cell (Cornillot et al. 2012); therefore, with parasitemia of 1–30 parasites per one 1 μl of blood, at least 2–60 copies of the *Babesia* 18S rRNA gene should be present in each microliter of blood. Since in the DNA isolation step, 1 ml of blood is converted into 100 μl of DNA solution, there should be around 20–600 copies of target sequence per microliter of the DNA solution. Therefore, mPCR-HRM sensitivity should be at least comparable to that of other PCR methods used for *Babesia* detection. However, we agree with the previously expressed opinion that direct comparison of the *Babesia* PCR detection methods is required for proper evaluation of their sensitivity (Wang et al. 2015b).

It should be emphasized that most existing PCR methods used for diagnosis of babesiosis in humans allow detection of single species infection—*B. microti* (Hildebrandt et al. 2013; Wang et al. 2015b; Wang et al. 2015a; Bloch et al. 2013; Rollend et al. 2013; Chan et al. 2013). In the USA, most infections are caused by *B. microti* while in Europe, most infections are caused by other *Babesia* species (Hildebrandt et al. 2013). The demand for multiplex PCR for detection of several *Babesia* species from blood has been expressed, and our mPCR-HRM method might be one of the possible ways to fill the demand (Hildebrandt et al. 2013). We experimentally confirmed the ability of mPCR-HRM to detect and differentiate at least three species of *Babesia* infecting humans: *B. microti*, *B. venatorum*, and *B. divergens*. The possibility of species determination could be important not only for epidemiology or basic research but also for medicine as due to possible differences in the clinical course of the infections caused by distinct *Babesia* species, different approaches towards treatment may be required (Ord and Lobo 2015).

In silico analysis showed that the primer B-rev was universal for at least 18 taxons of *Babesia* (*Babesia* sp. and *Babesia* species-like) (Table 2). The limited species specificity was achieved by application of forward primers, B-BM B-BDV, matching perfectly at least seven *Babesia* taxons (Table 2) including the five described to infect humans: *B. microti*, *B. microti-like*, *B. divergens*, *B. divergens-like*, *B. venatorum*. The DNA region spanned by those primers is polymorphic and relatively short (Fig. 1) which allows efficient amplification and differentiation of PCR products by HRM curve analysis (Fig. 2 and Fig. 3). The ability to differentiate *Babesia* spp. using HRM curves of PCR products was confirmed on samples containing DNA of *B. microti*, *B. divergens*, *B. venatorum*, and *B. canis* (Fig. 2 and Fig. 3).

Based on the test results obtained for *B. microti*, *B. divergens*, and *B. venatorum* and in silico analysis, it seems highly probable that *B. capreoli* and *B. odocoilei* which have the perfectly matching binding sites for primers used in mPCR-HRM will be amplified and their differentiation by curve shape will be possible. To the best of our knowledge, there have been no descriptions of human infections with these species of *Babesia*, but they could be within the scope of interests of biologists investigating wildlife.

In general, PCR amplification may occur even if some mismatches between the primers and the DNA template are present. Based on our experience with universal primers for filarial parasite detection in mosquitoes (Masny et al. 2016), we expected that detection of other *Babesia* species could be possible using mPCR-HRM with the designed primers. The DNA of *B. canis*, species specific for dogs, was amplified in the clinical samples of infected dogs by the primer pair B-BDV, B-rev, and HRM curve analysis allowed *B. canis* identification (Fig. S1). The ability to amplify *B. canis* DNA was perceived by us as an advantage—the range of detected *Babesia* species was broader than we initially expected to obtain. An experiment performed on clinical blood samples of ten dogs infected with *Babesia canis* showed reproducible results (Fig. S1) which confirmed that the mPCR-HRM could be applicable for laboratory diagnostics of canine babesiosis and that the presence of mismatches in one primer did not have a detrimental effect on the HRM curve-based species identification reproducibility. The reproducibility of HRM curve shapes was high also for *B. microti* DNA isolated from naturally infected *Microtus oeconomus* (Fig. S1).

Organisms currently classified as *Theileria* spp*.*, for example *Theileria* sp. HN1 (FJ645726.1), could be theoretically amplified by mPCR-HRM since it has perfect binding sites to our primers (Table S2). The classification to either *Theileria* or *Babesia* genus has been undergoing changes recently. Furthermore, classification to the same genus, *Theileria* or *Babesia*, might not reflect the real phylogenetic distance between organisms (Harris 2016). Thus, the mPCR-HRM test might be applicable to the piroplasms from both *Theileria* and *Babesia* genera which could be an advantage for researchers looking for new hosts of those parasites. Furthermore, *Babesia* taxons such as *Babesia* microti-like or *Babesia divergens*-like (also referred to as *Babesia microti*-related or *Babesia* cf. *microti*) are diverse and most probably include many separate species (Harris 2016; Baneth et al. 2015). Since the primers B-BDV, B-BM, and B-rev match sequences of multiple *Babesia microti*-like and *Babesia divergens*-like taxon representatives, it is difficult to tell how many *Babesia* species could be amplified with mPCR-HRM.

Screening of livestock for *Babesia* spp. revealed livestock infections with *Babesia* species not considered specific for the hosts in which those species were found. Based on the screening results, it was concluded that host specificity of *Babesia* was likely to be wider than it had been thought to be (Li et al. 2015). It cannot be excluded that a similar phenomenon might be observed in humans and using an assay detecting multiple *Babesia* species might lead to revealing piroplasm infections previously undescribed in people—new risk factors.

The combination of the ability to amplify a number of *Babesia* species and the low level of cross-reactivity with the host DNA (no cross-reactivity for the hosts tested here) could be desirable features in the screening studies. We have experimentally shown that mPCR-HRM was applicable to *Babesia* detection in different host samples, and we have not observed cross-reactivity with host DNA, i.e., tick, human, rodent, and deer. The latter is the host of *B. divergens* and *B. venatorum* infecting humans and recently is considered a likely reservoir of *B. venatorum* (Michel et al. 2014).

Other real-time assays using universal primers for *Babesia* species detection exist. For the survey of livestock pan-Babesia FRET-quantitative PCR was designed with a pair of universal primers and molecular probes capable to detect multiple species of *Babesia* with relatively low level of interspecies discrimination. Therefore, the final species differentiation step was sequencing (Li et al. 2015). In this respect, the pan-Babesia FRET-quantitative PCR assay is similar to other PCR assays with universal primers (Li et al. 2015; Bonnet et al. 2007). We deliberately chose HRM based identification due to the ability to differentiate known and unknown sequences and its lower costs compared to molecular probe based assays. The region spanned by the designed B-primers is polymorphic, and species-specific probes could be designed. Theoretically, the use of species-specific molecular probes might have the advantage of assuring higher specificity compared to mPCR-HRM at the price of lower range of detected parasites (with a single probe) and/or higher cost in the case of multiple *Babesia* species detection (multiple probes).

Amplification of *Babesia* DNA has been gaining popularity in detecting the parasites presence in the vectors of the disease—ticks (Venclikova et al. 2015; Orkun et al. 2014; Sytykiewicz et al. 2012; Mierzejewska et al. 2015). A strong positive association was found between the *B. microti* infection ratios in humans and ticks from the same territories (Diuk-Wasser et al. 2014). In our study, *Babesia* positive and negative tick DNA samples were selected based on the results of commonly used universal PCR with primer pair Bab-GF2 and Bab-GR2 followed by amplification with species specific primers and then by PCR product sequencing (Bonnet et al. 2007). Infected tick was detected and the species specific melting curve was obtained (manuscript in preparation). Instead of several steps (PCR, nested PCR, sequencing) (Mierzejewska et al. 2015), a single PCR HRM reaction for both detection and amplification might be applied to screening of ticks. Thus, mPCR-HRM could be a useful, time and resource saving tool for tick population screening for the assessment of the epidemiological risks associated to tick bite exposure.

The mPCR-HRM can be converted into multiplex PCR with detection of the products by gel electrophoresis. However, in such a version, the discrimination between species is limited to *B. microti* and the remaining ones (Fig. 4). This could be a solution for smaller laboratories where mPCR-HRM products could be sequenced only if the identification of the *Babesia* species was necessary. We have intended to present an inexpensive versatile tool for distinct types of screening studies; therefore, the protocols we applied to DNA isolation from various sources, i.e., blood, tissues of vertebrates, and from ticks, are similar and rely on the same set of reagents which can be prepared in-house (Boom et al. 1999; Masny et al. 2016). Apart from being cheaper, the in-house DNA isolation protocol may assure better sensitivity of the PCR detection compared to commercial kits (Schuurman et al. 2005).

To the best of our knowledge, PCR assay for detection and species identification of several *Babesia* species infecting humans and animals, in a single tube reaction, without application of molecular probes, has not been described to date. The range of detected *Babesia* species and lack of cross-reactivity with the DNA of various *Babesia* hosts, combined with relatively good sensitivity, might make mPCR-HRM a cost-effective tool for diagnostics and screening studies.

## Electronic supplementary material

Table S1 (DOCX 19 kb)

Table S2 (DOCX 20 kb)

Figure S1 (PDF 15 kb)
