# Supplementary material for: High-resolution melting PCR assay, applicable for diagnostics and screening studies, allowing detection and differentiation of several Babesia spp. infecting humans and animals
Source: Parasitol Res. 2017 Aug 10;116(10):2671–81. doi: 10.1007/s00436-017-5576-x (PMC5599466; doi:10.1007/s00436-017-5576-x)
Supplement: Supplementary file 1 — (DOCX 19 kb) [file 436_2017_5576_MOESM1_ESM.docx]

**Table S1. List of GenBank sequences used to design real-time PCR primers to detect *Babesia* 18S rRNA. Accesion numbers and the associated *Babesia* species / lineage are given.**

| *Babesia* species / lineage | Primer pairs | |
| --- | --- | --- |
|  | B-BM / B-rev | B-BDV / B-rev |
| *B. capreoli* |  | AY726010.1, FJ944827.1, FJ944828.1, GQ304526.1, HQ638134.1’ JF776403.1’ JF776404.1, JN543172.1, JN543173.1, JN543176.1, JN543177.1, JN543179.1, JN543184.1, JQ886061.1, JQ929918.1, JX083980.1, JX083983.1, JX627350.1, JX627353.1, X627354.1, JX627358.1, KC007121.1, KC007122.1, KJ465869.1, KM657248.1, KM657250.1, KP742785.1 |
| *B. divergens* |  | AB857845.1, AB857846.1, AB861504.1, AJ439713.1, AY046576.1, AY098643.2, AY144688.1, AY572456.1, AY648871.1, AY789076.1, EF458219.1, EF458220.1, EF458221.1, EF458223.1, EF458226.1, EF458227.1, EF458228.1, EU182594.1, EU182595.1, FJ944822.1, FJ944823.1, FJ944824.1, FJ944825.1, FJ944826.1, GQ304524.1, GQ304525.1, GU057385.1, HQ638136.1, JF776397.1, JF776398.1, JF776408.1, JN543174.1, JQ929916.1, JX083979.1, JX083981.1, JX083982.1, KC465973.2, KC465974.1, KC465975.2, KC465976.2, KC465977.2, KF447531.1, KP745627.1, U07885.1, U16370.1, Z48751.1 |
| *B. divergens*-like |  | KJ486559.1, JF776402.1, JF776401.1, JF776399.1 |
| *B. microti* | AB032434.1, AB050732.1, AB071177.1, AB083375.1, AB085191.1, AB112050.1, AB119446.1, AB190287.1, AB190435.1, AB190459.1, AB197940.1, AB219802.1, AB241631.1, AB241632.1, AB241633.1, AB242176.1, AB243677.1, AB243679.1, AB243680.1, AB576641.1, AB731747.1, AB736270.1, AF231348.1, AF231349.1, AY094354.1, AY144691.1, AY144692.1, AY144693.1, AY144694.1, AY144698.1, AY144700.1, AY144701.1, AY648882.1, AY693840.1, AY789075.1, AY943957.1, AY943958.1, EF413181.1, EU168705.1, GQ856649.1, GQ856652.1, GQ856653.1, GU057383.1, GU057386.1, JQ315417.1, JQ315420.1, JQ315422.1, JQ609304.1, JQ711225.1, JQ886033.1, JQ886035.1, JQ993429.2, JX417370.1, JX627356.1, JX679169.1, JX679172.1, JX962781.1, KC007119.1, KC007120.1, KC147722.1, KC147723.1, KC147724.1, KC470047.1, KC470048.1, KC470049.1, KC478600.1, KC581934.1, KC821597.1, KF410824.1, KF410825.1, KF410826.1, KF410827.1, KJ486556.1, KJ508857.1, KJ508858.1, KJ649287.1, KJ649288.1, KM610231.1, KP688578.1, KT318132.1, LC005752.1, LC005753.1, LC005754.1, LC005755.1, LC005757.1, LC005758.1, LC005759.1, LC005760.1, LC005761.1, LC005762.1, LC005763.1, LC005764.1, LC005765.1, LC005766.1, LC005767.1, LC005768.1, LC005769.1, LC005771.1, LC005772.1, XR_001160977.1, XR_001160982.1 |  |
| *B. microti*-like | KC904077.1, KC904078.1, KJ871352.1, KM115968.1, KM115972.1, KM115977.1, KM115985.1 |  |
| *B. odocoilei* |  | KC995286.1, KC995285.1, KC995283.1, KC460321.1, JX679176.1, AY046577.1, AY661510.1, AY661509.1, AY661507.1, AY661503.1, AY661502.1, U16369.2, AY144689.1 |
| *B. venatorum* (*Babesia* sp. EU1) |  | AY046575.1, AY648877.1, AY648879.1, DQ312434.1, DQ312436.1, EF185818.1, EF185819.1, FJ215872.1, GQ856650.1, GQ856655.1, GQ856656.1, GQ856657.1, GQ888709.1, GU647159.1, GU734773.1, HM113372.1, HM371184.1, HQ638133.1, HQ830266.1, JF776406.1, JF776407.1, JF776413.1, JF776415.1, JF922093.1, JN036420.1, JQ711228.1, JQ886024.1, JQ886029.1, JQ929917.1, JQ993425.2, JQ993428.2, JX040642.1, JX627357.1, KC007116.1, KC007117.1, KC007118.1, KF447532.1, KF500410.1, KF724377.1, KJ152840.1, KJ465867.1, KJ465868.1, KJ486557.1, KJ508859.1, KJ508860.1, KJ663730.1, KM095110.1, KM244044.1, KP072001.1, KP688579.1, KR003827.1, KR493901.1, KR493903.1, KR493904.1, KR493905.1, KR493906.1, KR493907.1, KR493908.1, LC005773.1, LC005775.1, LC005776.1, LC005777.1 |
