# Supplementary material for: High-resolution melting PCR assay, applicable for diagnostics and screening studies, allowing detection and differentiation of several Babesia spp. infecting humans and animals
Source: Parasitol Res. 2017 Aug 10;116(10):2671–81. doi: 10.1007/s00436-017-5576-x (PMC5599466; doi:10.1007/s00436-017-5576-x)
Supplement: Supplementary file 2 — (DOCX 20 kb) [file 436_2017_5576_MOESM2_ESM.docx]

**Table S2. *Theileria* sequences perfectly matching primers B-rev, B-BM, B-BDV.**

| Primer | Species based matching / similarity (%) | | | | | | | | | | | | |
| --- | --- | --- | --- | --- | --- | --- | --- | --- | --- | --- | --- | --- | --- |
|  | 1 | 2 | 3 | 4 | 5 | 6 | 7 | 8 | 9 | 10 | 11 | 12 | 13 |
|  | *T. annulata* | *T. buffeli* | *T. capreoli* | *T. cervi* | *T. equi* | *T. luwenshuni* | *T. orientalis* | *T. ornithorhynchi* | *T. ovis* | *T. sinensis* | *T. uilenbergi* | *T. velifera* | *Theiteria* sp. HN |
| B-rev | 23 / 23 (100%) | 23 / 23 (100%) | 23 / 23 (100%) | 23 / 23 (100%) | 22 / 23 (96%) | 23 / 23 (100%) | 23 / 23 (100%) | 23 / 23 (100%) | 23 / 23 (100%) | 23 / 23 (100%) | 23 / 23 (100%) | 23 / 23 (100%) | 23 / 23 (100%) |
| B-BM | 14 / 25 (56%) | 10 / 25 (40%) | 23 / 25 (92%) | 22 / 25 (88%) | 10 / 25 (40%) | 14 / 25 (56%) | 23 / 25 (92%) | 23 / 25 (92%) | 22 / 25 (88%) | 10 / 25 (40%) | 23 / 25 (92%) | 8 / 25 (32%) | 25 / 25 (100%) |
| B-BDV | 13 / 25 (52%) | 8 / 25 (32%) | 8 / 25 (32%) | 8 / 25 (32%) | 8 / 25 (32%) | 15 / 25 (60%) | 12 / 25 (48%) | 8 / 25 (32%) | 8 / 25 (32%) | 8 / 25 (32%) | 8 / 25 (32%) | 8 / 25 (32%) | 8 / 25 (32%) |

[1]: KU554731.1, KU510436.1, KT959231.1, KU714607.1, KT736499.1, KT736498.1, KT736497.1, KT736496.1, KT736495.1, KT356608.1, KT356607.1, KT356606.1, KT356605.1, KR184819.1;

[2]: KX115426.1, FJ225391.1, GU361798.1, GU361796.1, FJ225391.1, KU507512.1, KX115426.1, KJ850940.1, KJ850936.1, KJ715175.1, KJ715170.1, KJ806988.1, KJ806987.1, KJ806986.1, KJ183085.1, KJ183080.1,

[3]: KT725850.1, KU554727.1, KU554722.1, LC131102.1, LC131101.1, LC131100.1, LC131098.1, LC131093.1, LC131092.1, LC131081.1, LC131076.1, LC131070.1;

[4]: KT959227.1KT959225.1, KT959224.1, KT959223.1;

[5]: KU240068.1, KU240064.1, KT004408.1, KT004407.1, KJ801925.1, JX177672.1, JX177670.1, AB733373.2, EU642508.1, FJ209021.1, AY150062.2, AY150063.2, Z15105.1, KJ801922.1, XM_004828766.1, XM_004829632.1, CP001669.1, KJ573373.2, KU672386.1, KU240072.1, KU240069.1, KU240065.1, KP995259.1, KP177882.1, KP868758.1, KP868757.1, KJ801931.1, KJ801928.1, KM819520.1, KM046921.1, KM046920.1, KM046919.1, KM046918.1, KJ573370.1, KF559357.1, XM_004828834.1;

[6]:KU554730.1, KU554729.1, KU554726.1, KU554725.1, KU518032.1, KU518031.1, KU247949.1, KU247948.1, KU870897.1, KU356903.1, KT356604.1, KT356603.1, KT356602.1;

[7]: KU363043.1, KT725847.1, XM_009692225.1;

[8]: KT937392.1, KT937391.1, KT937390.1;

[9]: KU714608.1, KT851438.1, KT851437.1, KT851436.1, KT851435.1, KT851434.1, KT851433.1, KT851432.1, KT851431.1, KT851430.1, KT851429.1, KT851428.1, KT851427.1, KT851426.1, KT851425.1, KT851424.1, KR094869.1, KR094868.1, KR094867.1, KR094866.1, KR094865.1, KR094864.1, KR094863.1, KR094862.1, KR094861.1;

[10]: KX115427.1, KX115427.1, EU274472.1, EU277003.1, KM211713.1;

[11]: KU554729.1, KF781308.1, KF771184.1, KC778790.1, KC601647.1, KC601645.1, JF719835.1, AY262116.1, AY262122.1, AY262121.1, AY262120.1;

[12]: KU206307.1, KU206306.1, KU206305.1, KU206304.1, KU206303.1, KU206302.1, KU206301.1, KU206300.1, KU206299.1, KU206298.1;

[13]: FJ645726.1, FJ645725.1
