# Supplementary material for: High-resolution melting PCR assay, applicable for diagnostics and screening studies, allowing detection and differentiation of several Babesia spp. infecting humans and animals
Source: Parasitol Res. 2017 Aug 10;116(10):2671–81. doi: 10.1007/s00436-017-5576-x (PMC5599466; doi:10.1007/s00436-017-5576-x)
Supplement: Supplementary file 3 — (PDF 15 kb) [file 436_2017_5576_MOESM3_ESM.pdf]

Figure S1. Reproducibility of HRM curves.

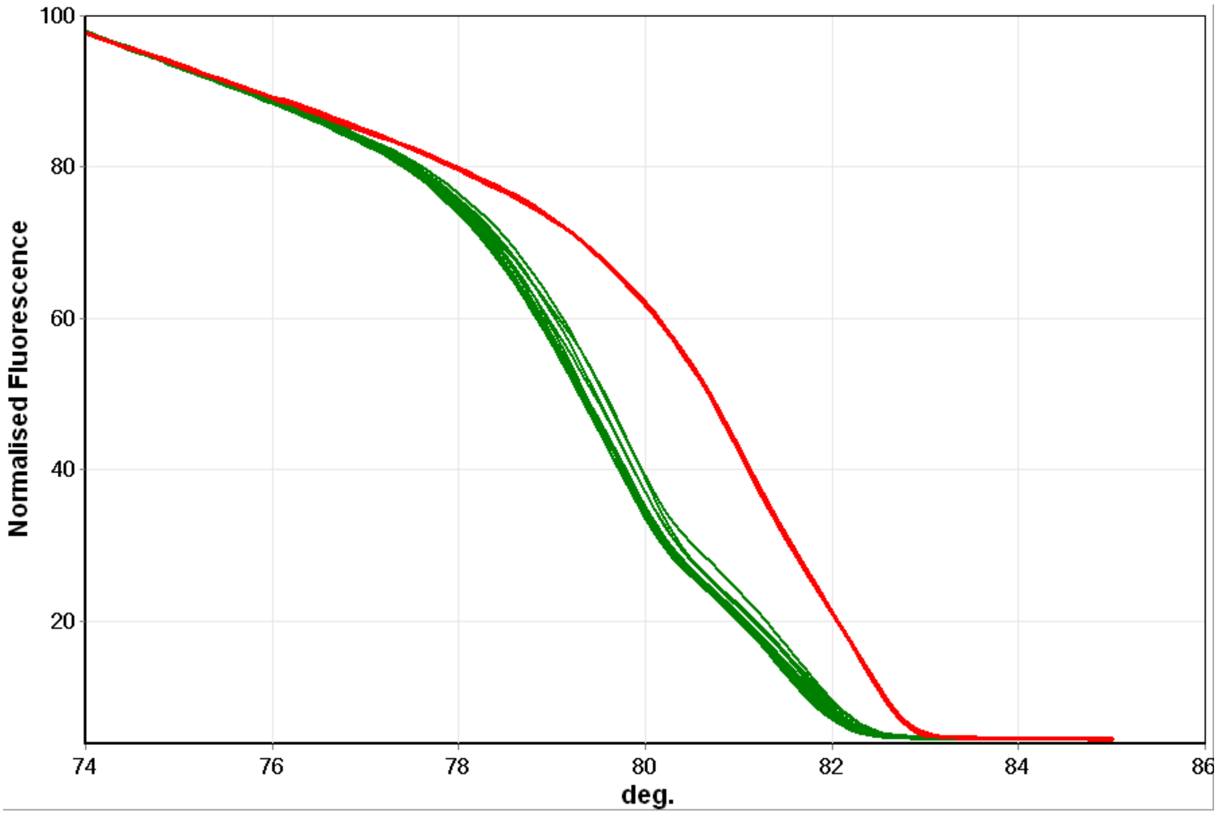

Red - melting curves of *B. microti* obtained in mPCR-HRM performed on DNA isolated from 5 distinct *Microtus oeconomus* blood samples.

Green - melting curves of *B. canis* obtained in mPCR-HRM performed on DNA isolated from 10 distinct *Canis canis* (dog) blood samples.
